# Molecular Detection of Spotted-Fever Group Rickettsiae in Ticks Collected from Domestic and Wild Animals in Corsica, France

**DOI:** 10.3390/pathogens8030138

**Published:** 2019-09-04

**Authors:** Vincent Cicculli, Oscar Maestrini, Francois Casabianca, Natacha Villechenaud, Remi Charrel, Xavier de Lamballerie, Alessandra Falchi

**Affiliations:** 1 Laboratoire de Virologie, Université de Corse-Inserm, 20250 EA7310 Corte, France; 2 Laboratoire de Recherches sur le Développement de l’Elevage (Research Laboratory for the Development of Livestock), 20250 UR045 Corte, France; 3 Unité des Virus Emergents (UVE: Aix Marseille Université, IRD 190, Inserm 1207, IHU Méditerranée Infection), 13000 Marseille, France

**Keywords:** ticks, Corsica, Rickettsia, wild animals, domestic animals, host

## Abstract

To obtain a better understanding of the current magnitude of tick-borne rickettsioses in Corsica, we used molecular methods to characterize the occurrence of *Rickettsia* spp. in ixodid ticks collected from domestic and wild animals. The presence of *Rickettsia* spp. was evaluated using real-time polymerase chain reaction targeting the *gltA* gene and by sequencing of *gltA* and *ompA* partial genes for species identification and phylogenetic analysis. Infection rates were calculated as the maximum-likelihood estimation (MLE) with 95% confidence intervals (CI). In total, 1117 ticks belonging to four genera (*Rhipicephalus*, *Hyalomma*, *Ixodes*, and *Dermacentor*) were collected from cattle, sheep, wild boars, and companion animals during July–August 2017 and July 2018–January 2019. Overall, *Rickettsia* DNA was detected in 208 of 349 pools of ticks (MLE = 25.6%, 95% CI: 22.6–28.8%). The molecular analysis revealed five different rickettsial species of the spotted-fever group (SFG). We highlighted the exclusive detection of *Candidatus* Ri. barbariae in *R. bursa* and of *Ri. aeschlimanii* in *H. marginatum.* *Rickettsia slovaca* was detected in *D. marginatus* collected from wild boars. This study provides the first evidence of the presence of *Ri. monacensis* in *I. ricinus* ticks isolated from a dog in Corsica. In conclusion, our data revealed wide dispersal of SFG Rickettsiae and their arthropod hosts in Corsica, highlighting the need for surveillance of the risk of infection for people living and/or working close to infected or infested animals.

## 1. Introduction

Rickettsiae are obligate intracellular Gram-negative bacteria that are transmitted to vertebrate hosts by arthropod vectors such as ticks, fleas, lice, and mites. The genus *Rickettsia* (family Rickettsiaceae; order Rickettsiales) comprises 31 species that cause diseases in vertebrate hosts, including humans and domestic and wild animals [1]. Ticks can transmit these bacteria to humans and animals by feeding. Members of the genus *Rickettsia* can be classified into four phylogenetic groups: (1) the spotted-fever group (SFG), (2) the typhus group (TG), which includes the agents of epidemic typhus and murine typhus, (3) the ancestral group (AG), consisting of *Ri. bellii* and *Ri. canadensis*, and (4) the transitional group, the *Ri. akari* group (TRG) [2]. The SFG and AG Rickettsiae are mainly associated with ticks, while TG and TRG Rickettsiae are associated with other arthropods such as lice, fleas, and mites [3].

*Rickettsia* spp. and their associated human clinical diseases vary depending on geographical location [4]. In Europe, rickettsioses are caused mainly by tick-borne SFG Rickettsiae [4]. In the Mediterranean area, Rickettsiae species have been detected in several tick species, but human rickettsioses are usually considered to be caused by *Ri. conorii*, the causative agent of Mediterranean spotted fever (MSF) [1,5]. *Rickettsia aeschlimanii*, *Ri. massiliae*, and *Ri. monacensis* can also cause a similar clinical presentation. Other species that have been associated with human disease and detected in Europe include *Ri. slovaca* and *Ri. raoultii*, both causes of tick-borne lymphadenopathy and *Dermacentor*-borne necrosis lymphadenopathy (TIBOLA/DEBONEL) [6,7].

Information about the circulation and distribution of Rickettsia-infected tick species in Corsica, a French Mediterranean island, is scarce and fragmented. *Rickettsia aeschlimanii* and *Candidatus* Ri. barbariae were identified in *Hyalomma marginatum* and *Rhipicephalus bursa* collected from domestic animals in 2016 [8]. *Rickettsia slovaca* has been detected in ticks collected from vegetation. *Rickettsia massiliae* was reported from an *R. turanicus* collected from a dog in Corsica in 2005, and *Ri. africae* was detected in one *Amblyomma variegatum* collected from bovine in August 2018.

To obtain a better understanding of the current magnitude of tick-borne rickettsioses in Corsica, we aimed to use molecular methods to describe the presence of tick-borne Rickettsiae in tick species collected from domestic and wild animals. From a public health perspective, a better understanding of local tick species and of *Rickettsia* species carried by them is essential to improve local surveillance systems.

## 2. Results

### 2.1. Sampling and Identification of Ticks

In total, 1117 ticks were collected from 1014 animals (381 cattle, 500 sheep, 119 wild boars, 13 dogs, and 1 cat) (Table 1). Of these, 1075 (96% of 1117) were adult ticks and 674 (60% of 1117) were male ticks (Table 1). The sequences of mitochondrial 16S rDNA fragments of 12 ticks selected in this study, were confirmed, after BLAST analysis, to be *D. marginatus* (*n* = 5), *R. bursa* (*n* = 1), *H. marginatum* (*n* = 1), *I. ricinus* (*n* = 1), and *R. sanguineus s.l.* (*n* = 4).

Overall, 834 ticks were collected from 381 cattle skins, from which, three tick species were morphologically identified. The most abundant species was *R. bursa* (*n* = 608; 73.0% of ticks collected in cattle) followed by *H. marginatum* (*n* = 216; 25.8%), and *I. ricinus* (*n* = 10; 1.2%) (Table 1).

Ten ticks were collected from 500 sheep in one farm (Biguglia, Haute-Corse) in June 2017. All these were *R. bursa* (Table 1).

Two-hundred forty-five ticks were collected from 119 wild boars from August 2018 to January 2019. Four tick species were identified in wild boars. The most abundant species was *D. marginatus* (*n* = 223; 91.0%), followed by *I. ricinus* (*n* = 13; 5.3%), *R. bursa* (*n* = 8; 3.3%), and *H. marginatum* (*n* = 1; 0.4%) (Table 1).

Twenty-seven ticks were collected from 13 dogs from July to August 2017. Two tick species were identified in dogs: *Rhipicephalus sanguineus s.l.* (*n* = 24; 88.8%) and *I. ricinus* (*n* = 3; 11.2%). One *D. marginatus* was collected from one cat.

### 2.2. Infestation Rate in Cattle, Sheep, and Wild Boars

**Figure 1:**
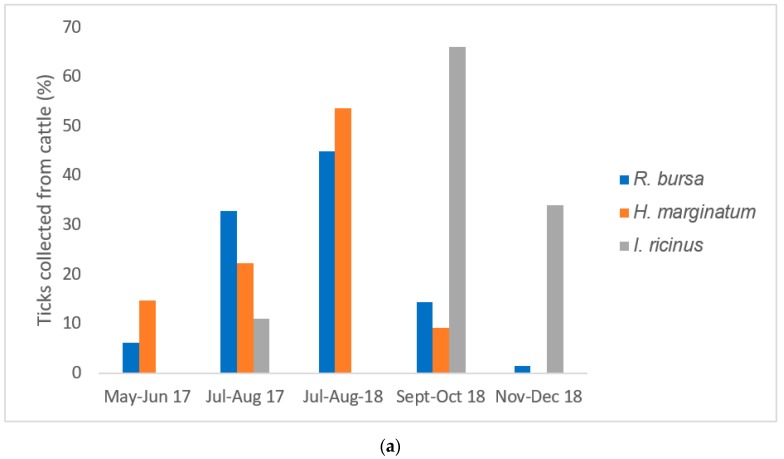

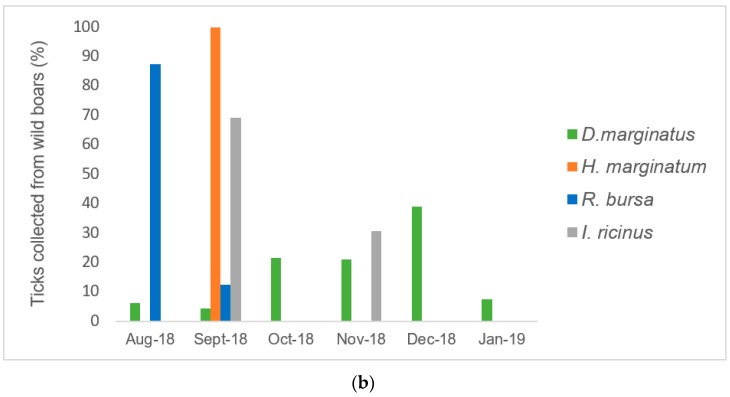
(**a**) Seasonal distribution of ticks collected from cattle (May–August 2017 and July–December 2018); (**b**) Seasonal distribution of ticks collected from wild boars (August 2018–January 2019).

The infestation rate in cattle was 19.2% (27/140) in 2017 (May–August 2017) and 36.0% (87/241) in 2018 (July–December 2018). Overall, the July–August 2018 infestation rate (65.1%; 54/83) was significantly higher than the July–August 2017 infestation rate (46.5%; 40/86) (*p* = 0.02), with a peak in July 2018 (70.7%; 29/41) (Figure 1a). The observed proportion of *H. marginatum* was significantly higher in July–August 2018 (53.7%; 116/216) than in July–August 2017 (22.2%; 48/216) (*p* < 0.0001) (Figure 1). Similarly, the observed proportion of *R. bursa* was significantly higher in July–August 2018 (45.0%; 208/608) than July–August 2017 (33.0%; 273/608) (*p* < 0.0001) (Figure 1a). *Ixodes ricinus* showed its peak activity in September–October 2018 (60.0%; 6/10).

The infestation rate of sheep was 2% and it was not drastically different from the 4% observed in cattle during the same period (May 2017) (*p* = 0.68).

The infestation rate observed in wild boars was 38.6% (46/119). *Dermacentor marginatus* was mainly collected from wild boars throughout the surveillance period (August 2018–January 2019), with a peak of activity in December 2018. September 2018 was characterized by the co-circulation of four species of ticks in wild boars (Figure 1b).

### 2.3. Detection of Rickettsial DNA

Overall, *Rickettsia* DNA was detected in 208 of 349 pools of ticks collected with an infection rate (maximum-likelihood estimation (MLE)) of 25.6% (95% CI: 22.6–28.8%). In ticks collected from cattle, *Rickettsia* spp. DNA was detected in 157 of 255 pools, with the highest infection rate for *H. marginatum* (50.5%, 95% CI: 37.0–64.4%) compared with *R. bursa* (16.6%, 95% CI: 13.3–20.4%) (*p* < 0.0001) and *I. ricinus* (2.7%, 95% CI: 0.1–11.6%) (*p* < 0.0001) (Table 2).

In sheep, the 10 ticks were analyzed in two pools. The detection rate of *Rickettsia* spp. DNA for *R. bursa* was 100%.

In ticks collected from wild boars, *Rickettsia* spp. DNA was detected in 46 of 78 pools with an infection rate of 24.4% (95% CI: 18.5–31.1%). The highest infection rate was observed for *D. marginatus* (23.0%, 95% CI: 16.9–30.0%) (Table 3).

The *Rickettsia* spp. DNA infection rate observed in ticks collected from cattle during July–August months of 2018 (34.7%, 95% CI 28.4–41.6%) was similar to that of 2017 during the same period (26.3%, 95% CI: 22.7–30.1%) (*p =* 0.219).

*Rickettsia* spp. DNA was detected in 2 (15.3%) of 13 tick pools collected from dogs. The infection rate was 5.0% (95% CI: 0.8–14.9%). The *D. marginatus* collected from one cat was also positive for *Rickettsia* spp. DNA.

### 2.4. Rickettsia Species Identification Based on Sequence Analysis

The molecular characterization of the Rickettsiae in ticks was based on *gltA* (*n* = 91 of 208 positive pools; 43.8%). A subset of positive samples (*n* = 33) was also characterized by sequencing *ompA* to confirm *Rickettsia* spp. (Table 4). The molecular analysis of *gltA* and *ompA* sequences revealed five different rickettsial species.

All *Rickettsia* detected in *H. marginatum* tick pools (*n* = 56) collected from cattle and wild boars were exclusively *Ri. aeschlimanii* (Table 4). *Candidatus* Ri. barbariae was the only bacterium detected in *R. bursa* tick pools collected from sheep (*n* = 2) and from cattle in 2017 (*n* = 5). The DNA of *Ri. aeschlimanii* and *Candidatus* Ri. barbariae were respectively detected in five and eleven *R. bursa* pools collected from cattle in 2018. The DNA of *Ri. slovaca* was detected in six *D. marginatus* tick pools, in two *I. ricinus* tick pools and in four *R. bursa* tick pools, all collected from wild boars. It is to be noted that *Ri. slovaca* DNA was also detected in one *D. marginatus* tick collected from a cat. *Rickettsia massiliae* and *Ri. monacensis* DNA have been detected in one pool of *R. sanguineus s.l.* ticks and in one pool of *I. ricinus* ticks collected from dogs.

### 2.5. Phylogenetic Analysis

**Figure 2:**
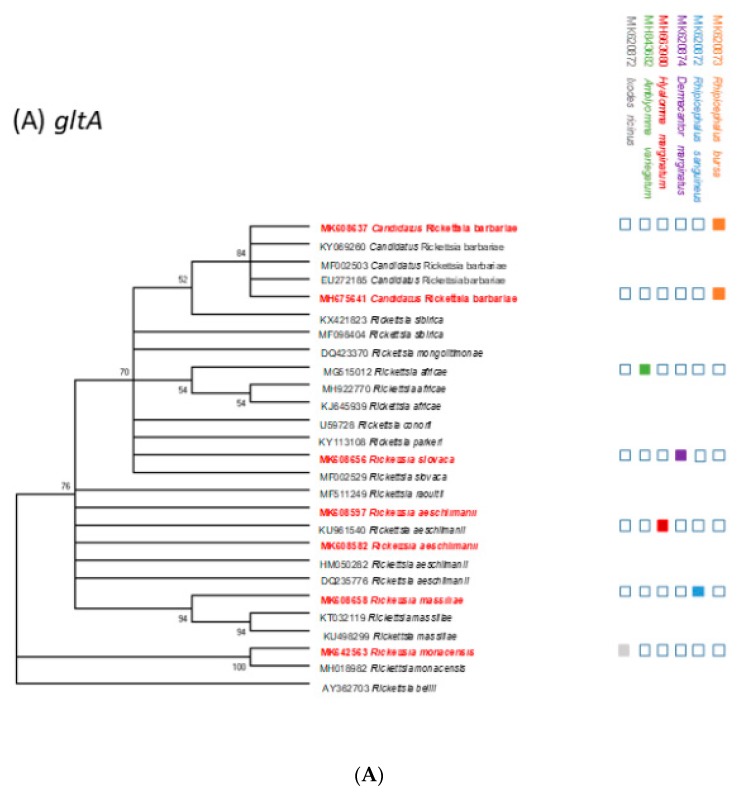

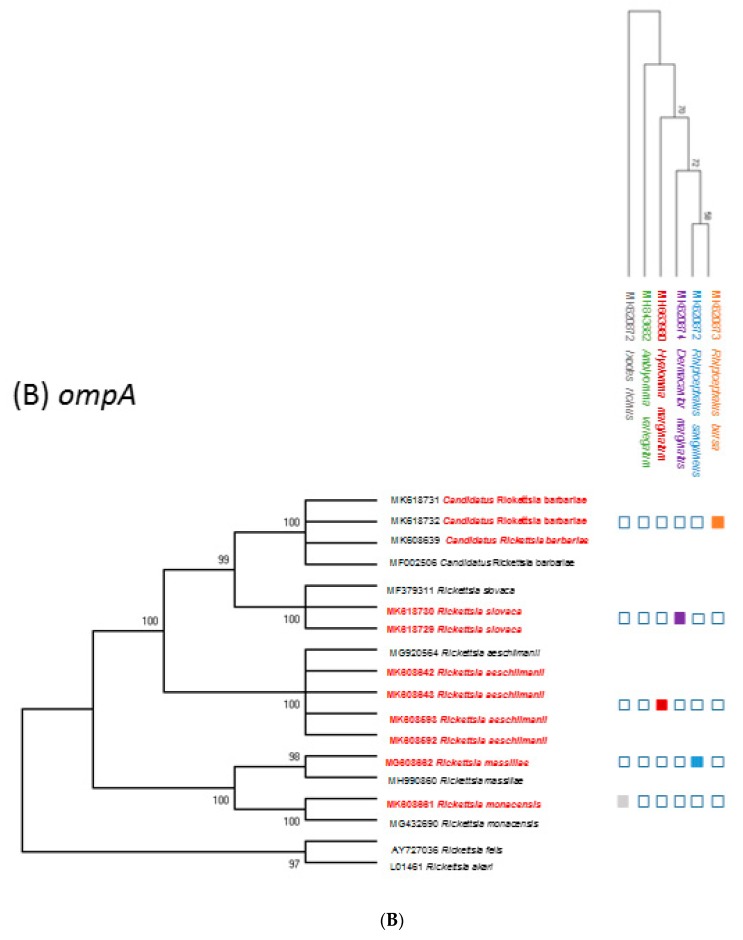
(**A**) A phylogenetic tree of spotted fever-group Rickettsine based on the gltA gene sequences. The analysis was performed using a maximum-likelihood method with the Kimura 2-parameter model. This analysis involved 27 nucleotide sequences. A II ambiguous positions were removed for each sequence pair. There were a total of 744 positions in the final dataset. The sequences detected in this study are indicated in red. The simlified tick phylogeny consisting of four species is indicated on the top right. The colored boxes indicate the presence of Rickettsia DNA in each tick species. (**B**) A phylogenetic tree of spotted-fever group *Rickettsiae* based on the *ompA* gene sequences. The analysis was performed using a maximum-likelihood method with the Kimura 2-parameter model. This analysis involved 18 nucleotide sequences. All ambiguous positions were removed for each sequence pair. There were a total of 493 positions in the final dataset. The sequences detected in this study are indicated in red. The simplified tick phylogeny consisting of four species is indicated on the top right. The colored boxes indicate the presence of *Rickettsia* DNA in each tick species.

The results of phylogenetic trees based on *gltA* and *ompA* sequences are illustrated in Figure 2A,B). Cluster I corresponded to *Ri. aeschlimanii*: the sequences were 100% identical to each other and to the *Ri. aeschlimanii* sequences deposited in GenBank (KU961540 (Russia) [*gltA*], MG920564 (Turkey) [*ompA*]). Cluster II represented the *Candidatus* Ri. barbariae group. Our sequences were 99–100% identical to each other and to the *Candidatus* Ri. barbariae detected in Sardinia (EU272185 [*gltA*] and MF002506 [*ompA*]). Cluster III represented *Ri. slovaca*; sequences were 100% identical to each other and showed 99.9% identity with that in GenBank (MF002529 (China) [*gltA*] and MF379311 (Turkey) [*ompA*]). Cluster four corresponded to the *Ri. monacensis* sequences group (100% identical to *Ri. monacensis* MH18982 (Poland) [*gltA*] and MG32690 (Italy) [*ompA*]). Cluster five included *Ri. massiliae* sequences and displayed 99.8% identity with *Ri. massiliae* (MH990860 (Pakistan) [*gltA*] and MH990860 (Pakistan) [*ompA*]).

## 3. Discussion

To our knowledge, this is the first reported evidence of the circulation of five bacteria from the genus of *Rickettsia* in four genera of ticks (*Rhipicephalus*, *Hyalomma*, *Dermacentor*, and *Ixodes*), collected from wild and domestic animals mainly reared in Northern Corsica.

In the present study, five tick species were collected, namely, *R. bursa*, *H. marginatum*, *I. ricinus*, *D. marginatus*, and *R. sanguineus s.l*. *Rhipicephalus* ticks were the most abundant species collected in this study. The diversity and seasonal pattern of ticks observed in this study, though collected not over 12 months and on selected animals kept under various conditions, is consistent with that reported previously for Corsica [8,9] and other Mediterranean countries [10,11,12]. In this study, overall, the low infestation rate observed in sheep with respect to cattle could be due to the short collection period and to a low density of tick populations usually reported in pastures where livestock had grazed with respect to undisturbed fallow lands [13].

In the present study, *R. bursa* was the tick species most frequently collected in cattle and sheep, with a peak of activity observed in the summer. This typical Mediterranean species is well established in the whole of Corsica [8,9]. In this study overall, almost 50% of *R. bursa* tick pools, mainly collected from cattle and sheep, were positive for *Rickettsia* spp. Interestingly, *Candidatus* Ri. barbariae DNA was exclusively detected in tick pools of *R. bursa* removed from sheep (100% of sequenced tick pools) and mostly in cattle (76%). Our detection of *Candidatus* Ri. barbariae in *R. bursa* collected from domestic animals reinforces its presence in Corsica ([8], pp. 606–613) and highlights the possible role played by *R. bursa* ticks in the natural maintenance of this bacterium. To understand better the role of *R. bursa* in the maintaining of *Candidatus* Ri. barbariae, ticks should be collected from nature. *Candidatus* Ri. barbariae is a presumed new species of the genus *Rickettsia* genetically characterized for the first time in *R. turanicus* from Sardinia [14], a Mediterranean island separated from Corsica by 10 km of sea. The presence of this bacterium has also been reported in several *Rhipicephalus* spp. in Portugal, Italy, France, Algeria, Cyprus, Israel, Cameroon, Lebanon [12,15,16,17,18,19], and in *Hyalomma* ticks in the West Bank [20]. The pathogenic role of *Candidatus* R. barbariae remains unknown for humans and animals, although it has been identified in an *R. bursa* tick removed from a woman in Greece [21].

*Hyalomma marginatum* was the second main tick species collected from cattle in the northern region of Corsica. This tick species was almost exclusively collected from cattle (99%). One *H. marginatum* was collected from a wild boar. *Rickettsia* spp. DNA was detected in more than 80% of *H. marginatum* pools collected from cattle sampling in Northern Corsica. *Hyalomma marginatum* ticks were exclusively infected with the human pathogen *Ri. aeschlimanii*, regardless *of* the host species (cattle or wild boars). In Corsica, cattle are reared outside all year round and acaricide treatments are rarely performed, increasing the opportunity of a wide circulation of this pathogen in Corsica. Such a high infection rate is consistent with the previously reported rate of infection of *Hyalomma* by *Ri. aeschlimanii* (>50% and >70%) in Corsica [8,22], in Croatia (64%) [23], and in Germany (50%) [24]. *Rickettsia aeschlimanii* may be spread through migratory birds from Africa, and it was detected in *Hyalomma* ticks collected from barn swallows (*Hirundo rustica*) in Corsica [25]. The first human case of infection was described in a patient who developed symptoms after returning from Morocco [26].

Even if wild boars and cattle share the same environment in Corsica [9], dominant tick species collected in the present study from these animals differed. In the present study, we confirmed that *D. marginatus* was the main tick species collected from wild boars, confirming the host preference previously reported. This tick species was not observed in cattle investigated in this study.

In the present study, most specimens of *D. marginatus* were collected during winter because the adults are active within a temperature range of 4–16 °C [27]. More than half of the pools of *D. marginatus* collected were positive for *Rickettsia* spp. Sequence analyses revealed for the first time the presence of *Ri. slovaca* DNA in *D. marginatus* ticks collected from wild boars in Corsica. This bacterium was previously detected in *D. marginatus* collected by flagging in Corsica and in other regions of the northern Mediterranean area. *Rickettsia slovaca* was identified in *D. marginatum* ticks collected on swine and wild boars in Sardinia [28]. Overall, these results suggested that the role of *D. marginatus* is important for the maintenance of *Ri. slovaca.* In Corsica, recreational hunting of wild boars could provide an ideal environment for the transmission of *Ri. slovaca* between wild boars, humans, and companion animals (*e.g.*, hunting dogs). At present, the *D. marginatus* tick is recognized as the main vector and reservoir for *Ri. slovaca* in Mediterranean areas, including southern Europe and North Africa [1]. This human pathogen has been detected in almost half of *D. marginatus* ticks collected from rodents in the northern Apennines (Italy) and by flagging in Kazakhstan [29,30]. *Rickettsia slovaca* is associated with a syndrome characterized by neck lymphadenopathy following tick bites [6]. Human infection with *Ri. slovaca* has been described in several European countries, including France [31]. We also reported for the first time the detection of *Ri. slovaca* DNA in one *D. marginatus* collected from a cat.

In this study, *R. sanguineus s.l.* ticks were exclusively collected from dogs. We reported the detection of *Ri. massiliae* DNA in one pool of *R. sanguineus s.l.* collected from a dog. *Rhipicephalus sanguineus* *s.l.* is the likely reservoir of *Ri. massiliae*, with transovarian passage rates up to 100%. *Rickettsia massiliae* was reported from a specimen of *R. turanicus* collected from a dog in Corsica [32] and has been putatively linked to mild to moderately severe illnesses in dogs in California [33]. *Rickettsia massiliae* is recognized as a pathogenic Rickettsia causing spotted fever in humans [1].

In the present study, *I. ricinus*, mainly collected during autumn, was sporadic and represented almost 2% of all ticks sampled. The DNA of *Ri. slovaca* was detected in pools of *I. ricinus* ticks collected from wild boars. We report for the first time the detection in Corsica of *Ri. monacensis* DNA in one pool of *I. ricinus* ticks collected from one dog. *Rickettsia monacensis*, an emerging human pathogen of the SFG Rickettsiae family, has been previously detected in *I. ricinus* collected from dogs in Spain [34] and in dog blood samples from the Maio Islands [35].

This study has some limitations. Finding the DNA of human and veterinary pathogens in feeding ticks is only a marker of the community of tick-transmitted pathogens circulating in the target territory [36]. Ticks were mostly analyzed in pooled samples and not individually. Working with pools can lead to underestimation of the infection rate, which must be interpreted with caution.

The ticks were mostly collected in a municipal slaughterhouse in Northern Corsica. We have no data about the southern part of the island. Moreover, ticks from cattle, wild boars, and dogs were collected from several locations while the sheep were only from one farm.

## 4. Methods

### 4.1. Study Area and Ticks

**Figure 3:**
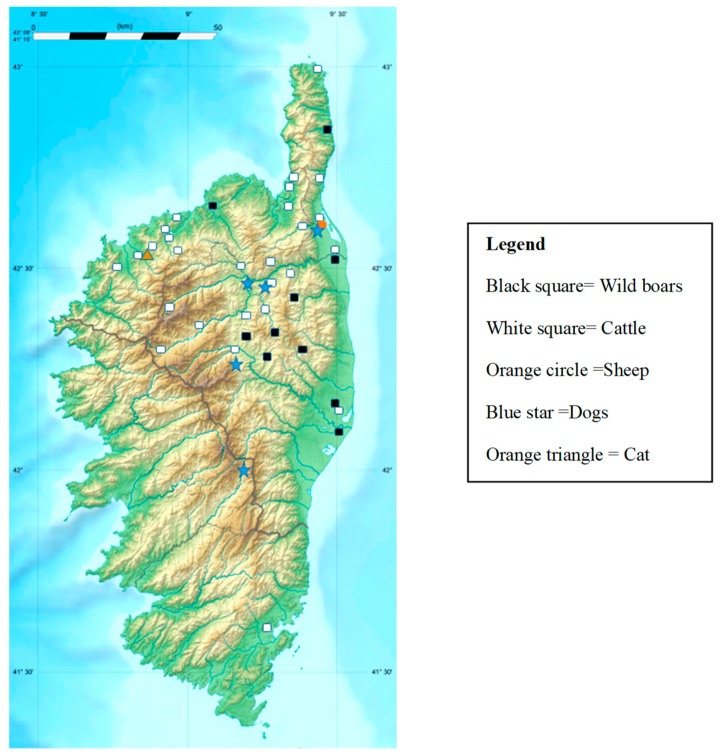
Map of Corsica, France, indicating the tick collection sites and the animal species. **GPS coordinates: Cattle**: Oletta (42°38′00″ N, 9°21′22″ E), Filicetu (42°32′40″ N, 8°56′09″ E), Corti (42°18′23″ N, 9°09′05″ E), Nessa (42°33′04″ N, 8°56′57″ E), Portivechju (41°35′30″ N, 9°16′49″ E), Omessa (42°22′16″ N, 9°12′39″ E), Lama (42°34′39″ N, 9°10′22″ E), Monticellu (42°37′05″ N, 8°57′16″ E), Lucciana (42°32′48″ N, 9°25′05″ E), Calinzana (42°30′31″ N, 8°51′21″ E), Lentu (42°31′22″ N, 9°16′57″ E), San lurenzu (42°23′06″ N, 9°17′28″ E), San Martinu di lota (42°43′26″ N, 9°27′21″ E), Casamaccioli (42°19′06″ N, 9°00′07″ E), Patrimoniu (42°41′54″ N, 9°21′44″ E), Olmeta-di-tuda (42°36′44″ N, 9°21′16″ E), Farinole (42°43′58″ N, 9°21′58″ E), Penta-Acquatella (42°27′55″ N, 9°21′49″ E), Zilia (42°31′52″ N, 8°54′06″ E), Castellu di rustinu (42°27′52″ N, 9°18′56″ E), Pietralba (42°32′51″ N, 9°11′11″ E), Santa-Reparata-di-Balagna (42°36′16″ N, 8°55′45″ E), Cateri (42°34′21″ N, 8°53′33″ E), Ruglianu (42°57′25″ N, 9°25′08″ E), Filicetu (42°32′40″ N, 8°56′09″ E), Ascu (42°27′16″ N, 9°01′59″ E), Corscia (42°21′20″ N, 9°02′36″ E), Vilone Ornetu (42°24′06″ N, 9°28′18″ E), Furiani (42°39′32″ N, 9°24′54″ E), Monte grossu (42°30′06″ N, 8°55′22″ E), Tallone (42°13′55″ N, 9°24′53″ E). **Wild boars:** Chiatra (42°17′34″ N, 9°28′34″ E), Tralonca (42°20′39″ N, 9°12′26″ E), Quercitellu (42°25′37″ N, 9°21′02″ E), Bustanicu (42°19′24″ N, 9°18′03″ E), Favalellu (42°17′43″ N, 9°16′20″ E), Venzolasca (42°29′06″ N, 9°27′26″ E), Sermanu (42°18′54″ N, 9°16′06″ E), Palasca (42°35′24″ N, 9°02′36″ E), Pianellu (42°17′26″ N, 9°21′39″ E), Cagnanu (42°52′34″ N, 9°25′50″ E), Santa-Lucia-di-Mercuriu (42°19′37″ N, 9°13′18″ E), Aleria (42°06′53″ N, 9°30′48″ E), Mazzola (42°18′05″ N, 9°18′40″ E). **Dogs**: Biguglia (42°37′41″ N, 9°25′14″ E), Casanova (42°15′19″ N, 9°10′30″ E), Castellu di Rustinu (42°27′52″ N, 9°18′56″ E), Ponte-Leccia (42°26′10″ N, 9°18′01″ E), Palneca 41°58′14″ N, 9°10′26″ E). **Cat**: Muru (42°32′47″ N, 8°54′54″ E). **Sheep**: Biguglia (42°37′41″ N, 9°25′14″ E).

Ticks were manually collected at several sites in Corsica (Figure 3) as follows: (i) in May 2017 from sheep (*n* = 500) bred in one farm located at the edge of the largest lagoon in Corsica, the Biguglia Nature Reserve; (ii) in July–August 2017 and July–December 2018 from cattle (*n* = 140 and *n* = 241, respectively) in the Ponte-Leccia slaughterhouse, and (iii) from wild boars (*n* = 119) during the 2018 hunting season (August 2018–January 2019). In July–August 2017, ticks were sampled from 13 dogs and one cat that presented at three veterinary clinics for a variety of reasons and not specifically for symptoms related to tick infestation.

Ticks were collected from sheep during milking mainly around the perineum and udder. The farmer declared he had never treated sheep against ectoparasites. The breeding system was extensive; thus, the sheep live in open pastures.

During each visit to the slaughterhouse, the whole skin of slaughtered animals was inspected, and ticks were collected manually. The national cattle identification system, which uses ear tags, allowed the origin of the animals to be tracked and the farm owners to be identified. One to 20 ticks were collected from each infested animal.

### 4.2. Morphologic Identification of Ticks at the Species Level

Ticks were identified at species level using a pictorial guide [37]. Specific attention was brought to *Ixodes* ticks to identify *I. inopinatus* [38].

### 4.3. DNA Extraction

Ticks were washed once in 70% ethanol for 5 min and then twice in distilled water for 1 min each time. Following species identification, ticks were analyzed individually or using monospecific pools consisting of two to six ticks according to developmental stage (nymphs, not engorged females, and male adults) and host. Individual or pools of ticks were crushed in phosphate-buffered saline using TissueLyser II (Qiagen, Hilden, Germany) at 5500 rpm for 20 s. DNA extraction was performed using QIAcube HT (Qiagen) and a QIAamp cador Pathogen Mini Kit according to the manufacturer’s instructions. DNA was eluted in 150 μL of buffer and stored at −20 °C. For each polymerase chain reaction (PCR) reaction, the template DNA was at a final amount of <200 ng.

### 4.4. Molecular Identification of Ticks at the Species Level

Morphological identification of ticks was confirmed using PCR amplification and sequencing of mitochondrial 16S rDNA [39] (Table 5).

### 4.5. PCR Detection of Rickettsia

The rPCR assays for *Rickettsia* spp. were performed using the primers and probes previously described for the amplification of the citrate synthase gene (*gltA*) (Table 5) [42]. Reactions were performed on a 96-well Applied Biosystems™ QuantStudio™ 3 Real-Time PCR System using QuantiFast Pathogen + Internal Control Kits (Qiagen). Internal and negative controls were included in each run.

Positive samples detected using rPCR were then analyzed using conventional PCR using primers that amplified the 850-bp fragment of the *gltA* gene common to the *Rickettsia* genus [40,41] and primers to amplify the 532-bp fragment of the 190-kDa outer membrane protein (*ompA*) gene specific to the SFG Rickettsiae [43] (Table 5). The reactions were carried out using an Applied Biosystems GeneAmp PCR System 9700 (Applied Biosystems, Courtaboeuf, France). The PCR products were separated on 2% agarose gels in tris-acetate-ethylenediaminetetraacetic acid (TAE buffer) and were visualized under ultraviolet light after staining with ethidium bromide. A 100-bp DNA ladder was used as a standard marker.

Strong positive samples were purified and directly sequenced using an Applied Biosystems model 3730XL (Fisher Scientific S. A. S., Illkirch-Graffenstaden, France). The newly generated sequences were aligned using Mega X [44]. All sequences were assembled and compared with similar sequences retrieved from the GenBank nucleotide database using BLASTn [45]. Phylogenetic analyses were inferred using the maximum-likelihood method implemented in the Mega X program [44].

### 4.6. Statistical Analysis

Infection rates for pooled ticks were calculated using the MLE method with 95% CI for unequal pool sizes and expressed as the MLE of infection rate per 100 ticks. MLE calculations are based on the number of pools, pool sizes (number of individuals per pool), and the number of positive pools [46].

The rates of infection or of tick infestation were compared using Fisher’s exact test (*p* < 0.05). The analysis was performed using the R statistical platform (version 3.1.2) [47].

## 5. Conclusions

In conclusion, our results contribute to a better knowledge about SFG Rickettsiae in Corsica and provide a useful contribution to understanding their epidemiology. This study reports the first evidence of the circulation of five species of the *Rickettsia* genus in Corsican ticks representing four genera, namely, *Rhipicephalus*, *Hyalomma*, *Dermacentor*, and *Ixodes*, that were collected from wild and domestic animals. We highlighted the exclusive detection of *Candidatus* Ri. barbariae in *R. bursa* collected from sheep and the wide circulation of the human pathogen *Ri. aeschlimanii* in *H. marginatum* collected from cattle. Moreover, the high percentage of *D. marginatus* with positive detection to *Ri. slovaca* in wild animals indicates a wide distribution of this human pathogen in Corsica. This study reports evidence for the first time that *Ri. monacensis*, a human pathogen, could circulate in ticks collected from dogs in Corsica. Implications for human health of the circulation of these two pathogens merits further investigation and should be taken into account by physicians seeing patients with unexplained febrile illness.

## 6. Declarations

### 6.1. Ethics Approval and Consent to Participate

The inspected cattle were slaughtered for human consumption. Living sheep were examined with the assistance of their owner. Companion animals were examined by veterinarians with the agreement of the owners. The wild boars collected were legally hunted during the hunting season.

### 6.2. Availability of Data and Materials 

The GenBank accession numbers obtained in this study were MK608560-MK608588; MK608594-MK608637; MK608656-MK608660; MK624992-MK624999; MK652441-MK652442 for *gltA* gene (*n* = 91) and MK608589-MK608593; MK608638-MK608655; MK618727-MK618732; MK609661; MK600862; MK608661-MK608662 and MK618727-MK618732 for *ompA* gene (*n* = 33). For tick identification (16S rDNA) the GenBank accession numbers obtained were: MK620874–MK620878 (*Dermacantor marginatus*), MK620873 (*R. bursa*), MH663977 for (H. marginatum) MK620872 (*I. ricinus*) and MK761069-MK761072 *(R. sanguineus s.l.)*. All other relevant data are included in the article.

## Figures and Tables

**Table 1:** Total tick species collected from hosts sampled.

Host (*n* of Hosts Inspected; Infestation Rate (%))	Tick Species	*n* Ticks (%)	Male *n* (%)	Female *n* (%)	Nymph *n* (%)
Cattle (*n* = 381; 37.7%)	*R. bursa*	608 (73.0)	350 (57.6)	225 (37.0)	33 (5.4)
	*H. marginatum*	216 (25.8)	163 (75.4)	53 (24.6)	0 (0.0)
	*I. ricinus*	10 (1.2)	3 (3.0)	7 (7.0)	0 (0.0)
	Total	834	516 (62.0)	285 (34.0)	33 (4.0)
Sheep (*n* = 500; 2.0%)	*R. bursa*	10 (100)	10 (100.0)	0	0
	Total	*10*	*10*		
Wild boars (*n* = 119; 38.6%)	*D. marginatus*	223 (91.0)	144 (64.5)	79 (35.5)	0 (0.0)
	*I. ricinus*	13 (5.3)	1 (8.0)	12 (92.0)	0 (0.0)
	*R. bursa*	8 (3.3)	1 (12.5)	7 (87.5)	0 (0.0)
	*H. marginatum*	1 (0.4)	0 (0.0)	1 (100)	0 (0.0)
	Total	*245*	*146 (60.0)*	*99 (40.0)*	*0 (0.0)*
Dogs (*n* = 13 * ^§^)	*R. sanguineus s.l.*	24 (89.0)	2 (8.3)	14 (58.3)	8(33.3)
	*I. ricinus*	3 (11.0)	0	3 (100)	0
	Total	*27*	*2 (7.4)*	*17 (63.0)*	*8 (29.6)*
Cat (*n* = 1 * ^§^)	*D. marginatus*	1 (100)	0	0	1(100)
Total (*n* = 1014)	All species	*1117*	*674 (60.4)*	*401 (35.8)*	*42 (3.8)*

* Tick collected by veterinarian. ^§^ infestation rate not estimable.

**Table 2:** Distribution of detected *Rickettsia* spp. in the tick species collected from cattle in 2017 (May–August 2017) and 2018 (July–December 2018) surveillance periods.

Number of Individual Ticks or Ticks per Pool (*n*)	Number of Pools with *n* Ticks				Positive *Rickettsia* spp. *n* s(%)			
2017 (July–August)	***H. marginatum***	***R. bursa***	***I. ricinus***	***Total***	***H.marginatum***	***R.bursa***	***I. ricinus***	**Total**
1	15	19	1	35	11 (73)	6 (32)	0 (0)	17 (49)
2	13	9	0	22	11 (85)	1 (11)	0 (0)	12 (55)
3	6	6	0	12	4 (67)	1 (17)	0 (0)	5 (42)
4	1	7	0	8	1 (100)	3 (43)	0 (0)	4 (50)
5	1	8	0	9	1 (100)	5 (63)	0 (0)	6 (67)
6	2	19	0	21	1 (50)	8 (42)	0 (0)	9 (43)
Total pools	38	68	1	107	29 (76)	24 (35)	0 (0)	53 (50)
MLE (95% CI)					50.5% (37.0–64.4)	12.2% (8.1–17.4)	0	20.7% (16.0–26.1)
2018 (July–December)	***H. marginatum***	***R. bursa***	***I. ricinus***	***Total***	***H. marginatum***	***R. bursa***	***I. ricinus***	**Total**
1	25	12	1	38	24 (96)	5 (42)	0 (0)	29 (76)
2	9	7	1	17	5 (55)	5 (71)	0 (0)	10 (62)
3	14	8	1	23	13 (93)	4 (50)	1 (100)	18 (78)
4	5	15	0	20	4 (80)	7 (46)	0 (0)	11 (55)
5	5	14	0	19	5 (100)	10 (66)	0 (0)	15 (79)
6	1	30	0	31	1 (100)	20 (64)	0 (0)	21 (68)
Total pools	59	86	3	148	52 (88)	51 (58)	1 (33)	104 (70)
MLE (95% CI)					65.3% (52.8–77.3)	20.1% (15.3–25.6)	2.8% (0.1–11.9)	30.7% (25.6–36.2)
2017 and 2018	***H. marginatum***	***R. bursa***	***I. ricinus***	***Total***	***H. marginatum***	***R. bursa***	***I. ricinus***	**Total**
1	40	31	2	73	35 (87)	11 (35)	0 (0)	46 (63)
2	22	16	1	39	16 (73)	6 (37)	0 (0)	22 (56)
3	20	14	1	35	17 (85)	5 (36)	1 (100)	23 (66)
4	6	22	0	28	5 (83)	10 (45)	0 (0)	15 (53)
5	6	22	0	28	6 (100)	15 (68)	0 (0)	21 (75)
6	3	49	0	52	2(67)	28 (56)	0 (0)	30 (58)
Total pools	97	154	4	255	81 (83)	75 (48)	1 (25)	157 (61)
MLE (95% CI)					58% (49.4–67.9)	16.6% (13.3–20.4)	2.7% (0.1–11.6)	26.3% (22.7–30.1)

**Table 3:** Distribution of detected *Rickettsia* spp. in the tick species collected from wild boars (August 2018–January 2019).

Number of Individual Ticks or Ticks per Pool (*n*)	Number of Pools with *n* Ticks					Positive *Rickettsia* spp. *n* (%)				
	*D. marginatus*	*I. ricinus*	*R. bursa*	*H. marginatum*	Total	*D. marginatus*	*I. ricinus*	*R. bursa*	*H. marginatum*	Total
1	15	1	3	1	20	8 (53)	1 (100)	2 (67)	1 (100)	12 (60)
2	10	2	1	0	13	7 (70)	1 (50)	1(100)	0 (0)	9 (62)
3	11	0	1	0	12	7 (64)	0 (0)	1(100)	0 (0)	8 (67)
4	11	2	0	0	13	8 (73)	0 (0)	0 (0)	0 (0)	8 (62)
5	9	0	0	0	9	3 (34)	0 (0)	0 (0)	0 (0)	3 (33)
6	11	0	0	0	11	6 (56)	0 (0)	0 (0)	0 (0)	6 (55)
Total pools	67	5	5	1	**78**	39 (58)	2 (40)	4 (80)	1 (100)	46 (59)
**MLE (95% CI)**						23.0% (16.9–30.0)	4.9% (0.0–14.4)	10.9% (3.5–23.7)	2.4% (0.0–10.2)	24.4% (18.5–31.1)

**Table 4:** The spotted-fever group Rickettsiae identified in tick species collected from different hosts.

Host	Tick Species	No. of Pools Positive for Rickettsiae	No of *gltA* Sequences	No of ompA Sequences	Identified *Rickettsia* Species
2017					
Cattle	***H. marginatum***	29	24	*5*	*Ri.* *aeschlimanii*
	***R. bursa***	24	5	*2*	*Candidatus* Ri. barbariae
Sheep	***R. bursa***	2	2	*1*	*Candidatus* Ri. barbariae
Dogs	***R.*** ***sanguineus*** ***s.l.***	1	1	*1*	*Ri. massiliae*
	***I. ricinus***	1	1	*1*	*Ri. monacensis*
Cat	***D. marginatus***	1	1	*0*	*Ri. slovaca*
Total pools 2017		***58***	***34***	***10***	
2018					
Cattle	***H. marginatum***	52	31	*13*	*Ri.* *aeschlimanii*
	***R. bursa***	51	14	*5*	*Ri. aeschlimanii; Candidatus* Ri. barbariae
	***I. ricinus***	1	1	*0*	*Ri.* *aeschlimanii*
Wild boars	***D. marginatus***	39	6	*3*	*Ri. slovaca*
	***I. ricinus***	2	2	*0*	*Ri. slovaca*
	***R. bursa***	4	2	*2*	*Ri. slovaca*
	***H. marginatum***	1	1	*0*	*Ri.* *aeschlimanii*
Total pools 2018		***150***	***57***	***23***	
**Total pools 2017** **–** **2018**		***208***	***91***	***33***	***Ri. aeschlimanii; Candidatus* Ri. barbariae; *Ri. massiliae; Ri. monacensis; Ri. slovaca***

**Table 5:** Primers and probes used in this study.

Species	Target	Name	Sequence	Annealing Temperature (°C)	References
	**rPCR**				
*Rickettsia* spp.	*gltA*	*Rspp-F*	GAGAGAAAATTATATCCAAATGTTGAT	60	
		*Rspp-R*	AGGGTCTTCGTGCATTTCTT		
		*Rspp-P*	CATTGTGCCATCCAGCCTACGGT		
	**Conventional PCR (Sequencing)**				
*Rickettsia* spp.	*gltA*	*CS2D*	ATGACCAATGAAAATAATAAT	54	[40,41]
		*CSEndR*	CTTATACTCTCTATGTACA		
		*409D*	CCTATGGCTATTATGCTTGC	54	
		*1258R*	ATTGCAAAAAGTACAGTGAACA		
	*ompA*	*Rr190.70p*	ATGGCGAATATTTCTCCAAAA	48	
		*Rr190.602n*	AGTGCAGCATTCGCTCCCCCT		
Ticks	16S rDNA	*16S* + *1*	CTGCTCAATGATTTTTTAAATTGCTGTGG	48 and 54	[39]
		*16S* − *1*	CCGGTCTGAACTCAGATCAAGT		

## Abbreviations

**DEBONEL**	*Dermacantor*-borne necrosis lymphadenopathy
***gltA***	citrate synthase
**MLE**	maximum likelihood estimation
**MSF**	Mediterranean spotted fever
***ompA***	outer membrane proteins A
**PCR**	polymerase chain reaction
**SFG**	spotted fever group
**TIBOLA**	tick-borne lymphadenopathy
